# Enhanced hydrogen evolution from CuO_x_-C/TiO_2_ with multiple electron transport pathways

**DOI:** 10.1371/journal.pone.0215339

**Published:** 2019-04-15

**Authors:** Xiuying Huang, Meng Zhang, Runze Sun, Gaoyuan Long, Yifan Liu, Weirong Zhao

**Affiliations:** Department of Environmental Engineering, Zhejiang University, Hangzhou, China; University of California Santa Barbara, California, USA

## Abstract

Titanium dioxide nanoparticles co-modified with CuO_*x*_ (0≤x≤2) and carbonaceous materials were prepared with a simple hydrolysis and photo-reduction method for photocatalytic hydrogen generation. SEM/TEM and XPS analysis indicated that the carbonaceous materials were mostly coated on the TiO_2_ surface and clearly revealed that the Cu species exhibited multivalence states, existing as CuO_*x*_ (0≤x≤2). The optimal catalyst showed a 56-fold enhanced hydrogen evolution rate compared with that of the pure C/TiO_2_ catalyst. Further, an intensive multiple electron transfer effect originating from CuO_*x*_ and the carbonaceous materials is proposed to be responsible for the elevated photoactivity. CuO_*x*_ species serve as electron donors facilitating charge carrier transfer and proton reduction sites. The carbonaceous materials function as the “bridge” that transfers the electrons of TiO_2_ to the CuO_*x*_ species, which provides a new route for electron transfer and reinforces the effect of CuO_*x*_ as a co-catalyst. In this study, the CuO_*x*_ and C co-modified TiO_2_ catalyst was prepared with multiple electron transport pathways and enhanced hydrogen production evolution, which provides a deep understanding for the design of co-catalyst-based photocatalysts.

## 1 Introduction

Hydrogen, as an efficient, renewable and clean energy source, is considered to be one of the best fuels for the future [1, 2]. Hence, photocatalytic hydrogen production with semiconductors has been regarded as a promising strategy for its mild reaction conditions, and cost-effectiveness [3, 4]. Among the various studied photocatalysts, titanium dioxide (TiO_2_) has received tremendous attention for photocatalytic hydrogen evolution owing to its suitable conduction and valence bands edge position, as well as its characteristics of nontoxicity, low cost, and photocorrosion resistance [5, 6]. However, the undesirable charge carrier recombination and the inferior solar light utilization efficiency of TiO_2_, which leads to the low photocatalytic hydrogen generation efficiency, restrict their photochemical applications [7]. It is a hot issue to develop an effective method to overcome the intrinsic limitation.

Co-catalysts [8–10] have been developed to enhance charge carrier separation and activate redox reactions on catalyst surfaces. Noble metals such as Pt [11] and Pd [12] are often used as co-catalysts for TiO_2_, increasing the hydrogen evolution rate significantly. Noble metals can accept electrons from a semiconductor due to its high work function, which facilitates electron-hole separation. Their high-activity surfaces can act as active sites for hydrogen evolution. However, noble metals are hardly employed for practical applications due to their scarcity and valuableness.

As an alternative, transition metals (e.g., Ni, Cu, Co) [13–15] have emerged as an effective co-catalyst for H_2_ evolution. Among them, copper is one of the most promising candidates [16–18]. Ohno et al. [19] prepared TiO_2_ modified with Cu and other ions (iron(III), nickel(II) and chromium(III) ions), revealing that metal ions can serve as both electron acceptors and donors based on a double-beam photoacoustic spectroscopy measurement. Characterization experiments and theoretical calculations confirmed this conclusion [20]. In addition, Nosaka et al. [21] proposed an interfacial charge transfer (IFCT) mechanism for the Cu/TiO_2_ in which transition metal ions can function as a trap for excited electrons to decrease recombination. The authors believed that Cu(I) can be formed by the photoexcited electron reduction of Cu(II), and Cu(II)-grafted samples are capable of reducing O_2_ to O_2_^**∙-**^. The multivalent states of Cu are regarded as the active species for the photoreaction. Our group fabricated Cu(II) nanodot doped nanosheet TiO_2_ in a previous report [22], in which H_2_ evolution increased 25-fold due to the multiple charge-transfer pathways. Here, we introduced another species co-modified with Cu and managed to raise the hydrogen quantity.

Carbonaceous materials, such as carbon nanotubes (CNTs) [23] and graphene [24], have been considered as another kind of efficient co-catalyst for photocatalytic applications. Xiang et al. [25] prepared graphene-modified titania nanosheets and a 41-time enhancement of H_2_ evolution rate was observed. The authors revealed that graphene can facilitate electron transfer by accepting photoelectrons as well as functioning as active sites for the photocatalytic reaction. A similar mechanism is also applicable to CNT-TiO_2_ composites [26].

TiO_2_ co-modified with transition metals and nonmetal carbonaceous materials is a new route to facilitate the charge carrier separation and increase photoactivity. Zhang et al. [27] prepared a Cu-grafted TiO_2_/graphene photocatalyst for efficient phenol degradation, in which graphene provides a new electron transfer route and enhances the co-catalyst effect for superoxide radical generation.

Inspired by the above achievements, we have prepared a foam-like CuO_*x*_-C/TiO_2_ catalyst via a facile hydrolysis and photo-reduction method for photocatalytic hydrogen generation. By controlling the amount of water, the titanium precursor can be hydrolyzed completely. Thus, the carbonaceous materials originated from the calcination process without an external carbon precursor. The formation of carbonaceous materials and Cu species were systematically explored. The results demonstrated that carbonaceous materials serve as electron conductors to enhance the co-catalyst effect of CuO_*x*_, inducing multiple electron transfer pathways for photocatalytic hydrogen generation.

## 2 Experiment section

### 2.1 Materials

Tetrabutyl titanate (Ti(OBu)_4_), copper chloride (CuCl_2_.2H_2_O), ethanol (C_2_H_5_OH), and glycerol (C_3_H_8_O_3_) were purchased from Sinopharm Chemical Reagent Co., Ltd, China. All reagents were analytical grade and used without further purification. Deionized water was used for all experiments.

### 2.2 Catalyst preparation

C/TiO_2_ (CT) photocatalysts were synthesized by a facile sol-gel method. Typically, 5.5 mL of deionized water was added to 25 mL of tetrabutyl titanate (Ti(OBu)_4_) under magnetic stirring for 48 h. The mixture was filtered by a Buchaer funnel and dried in an oven at 80°C overnight. The prepared catalyst was denoted as CT. Then, CT samples were calcined at 350°C, 400°C, 500°C and 550°C for 2 h, respectively. The corresponding dark brown powders were denoted as CTR (R = 350, 400, 500 and 550).

CuO_*x*_-C/TiO_2_ was prepared according to a previously published photo-reduction method[28]. A certain amount of CTR catalyst was added to 100 mL of solution containing 5 v% glycerol and a given volume of CuCl_2_ aqueous solution (0.01mol/L). The mixture was placed to a photocatalytic quartz reactor which was bubbled with Ar for 0.5 h and then irradiated with a 300 W xenon lamp for 2 h under magnetic stirring. After extensive washing, the precipitate was dried at 80°C overnight and then calcined at 350°C with a heating rate of 5°C min^-1^ for 2 h. The prepared powders were denoted as CuO_*x*_-CTR (R = 350, 400, 500 and 550), the Cu/Ti molecule ratio of which is 1%.

### 2.3 Characterization

The structural characterization of the as-synthesized materials was performed by X-ray diffraction (XRD, PANalytical, Netherlands), and their morphology of them was observed by a scanning electron microscope (SEM, Supra55, Zeiss, Germany), transmission electron microscope (TEM, JEM-2010, JEOL, Japan), and high-resolution TEM, as illustrated in a previous work [22]. Molybdenum support films were used instead of carbon support films for TEM sample preparation. Element analysis (EA), X-ray photoelectron spectroscopy (XPS, Escalab 250Xi, Thermo, England) and UV–vis diffuse reflectance spectroscopy (UV–vis DRS, TU-1901, Pgeneral, Beijing, China) were used to monitor the components and surface chemical states of the prepared photocatalysts. The crystallization behavior was recorded using a NETZSCH STA490PC TG-DSC instrument with the temperature ranging from room temperature to 900°C. Specific surface area of the photocatalysts were measured by the Brunauer–Emmett–Teller (BET) method with automatic analyzer (3H-2000PS2, Beishide, Beijing, China) and the pore-size distributions were determined by using absorption curves and the Barrett−Joyner−Halenda (BJH) method.

To explore the charge carriers, photoluminescence emission spectra were measured at room temperature with a fluorescence spectrophotometer (FLS920, Edinburgh, England) using a 325 nm wavelength laser as the excitation source. In addition, all photoelectrochemical measurements (photocurrent densities vs time curves I–t, Nyquist plots and Tafel spectra) were conducted with a three-electrode workstation (CHI 660D, Shanghai, China).

### 2.4 Photocatalytic activity experiment

The photocatalytic activity experiment was performed in a top-irradiation jacketed quartz photoreactor with water flowing through to maintain a constant temperature (30 ± 1°C). In a typical reaction, 50 mg of photocatalyst was dispersed in 100 mL of 5 v% glycerol with magnetic stirring. The catalyst suspension was first degassed with an Ar stream for an hour. Subsequently, the solution inside the reactor was irradiated by a 300 W xenon lamp (CEL-HXUV300, Aulight, Beijing, China). The generated H_2_ was detected every half hour by a gas chromatograph (Fuli 9790, Zhejiang, China) equipped with a thermal conductivity detector using Ar as carrier gas. Another gas chromatograph with a flame ionization detector (Fuli 9790, Zhejiang, China) was used to determine the evolution of CO and CO_2_.

## 3 Results and discussions

### 3.1 Morphology and crystal structure

**Fig 1:**
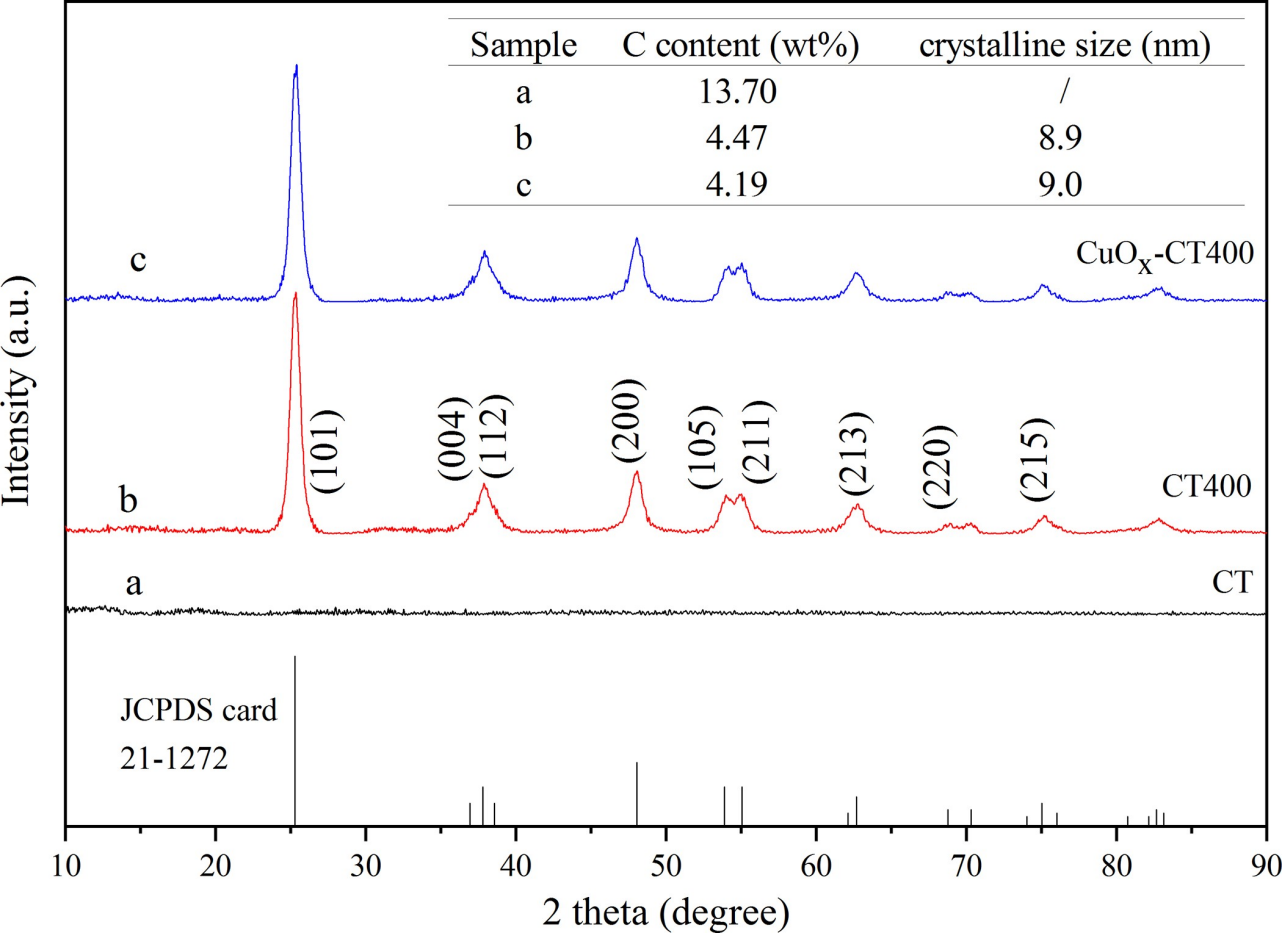
XRD patterns of the studied samples.

X-ray powder diffraction was used to analyze the crystal structures of CT, CT400 and CuO_*x*_-CT400. As shown in Fig 1, the XRD pattern of the CT sample without thermal treatment shows broad amorphous features and no peaks indicative of crystalline phases. After a 400°C treatment for 2 h, the CT400 exhibits characteristic peaks at 25.281°, 37.800°, 38.575°, 48.050°, 53.890°, 55.060°, 62.121°, 70.311°, and 75.032° corresponding to anatase TiO_2_ (JCPDS 21–1272) [22]. After CuO_*x*_ modification, the spectrum exhibits a similar peak shape, suggesting that the addition of Cu species did not change the crystal form. Moreover, the average grain size of CT400 was estimated to be approximately 9 nm by using Jade 6.0 and Scherrer’s formula [29] from the XRD data.

**Fig 2:**
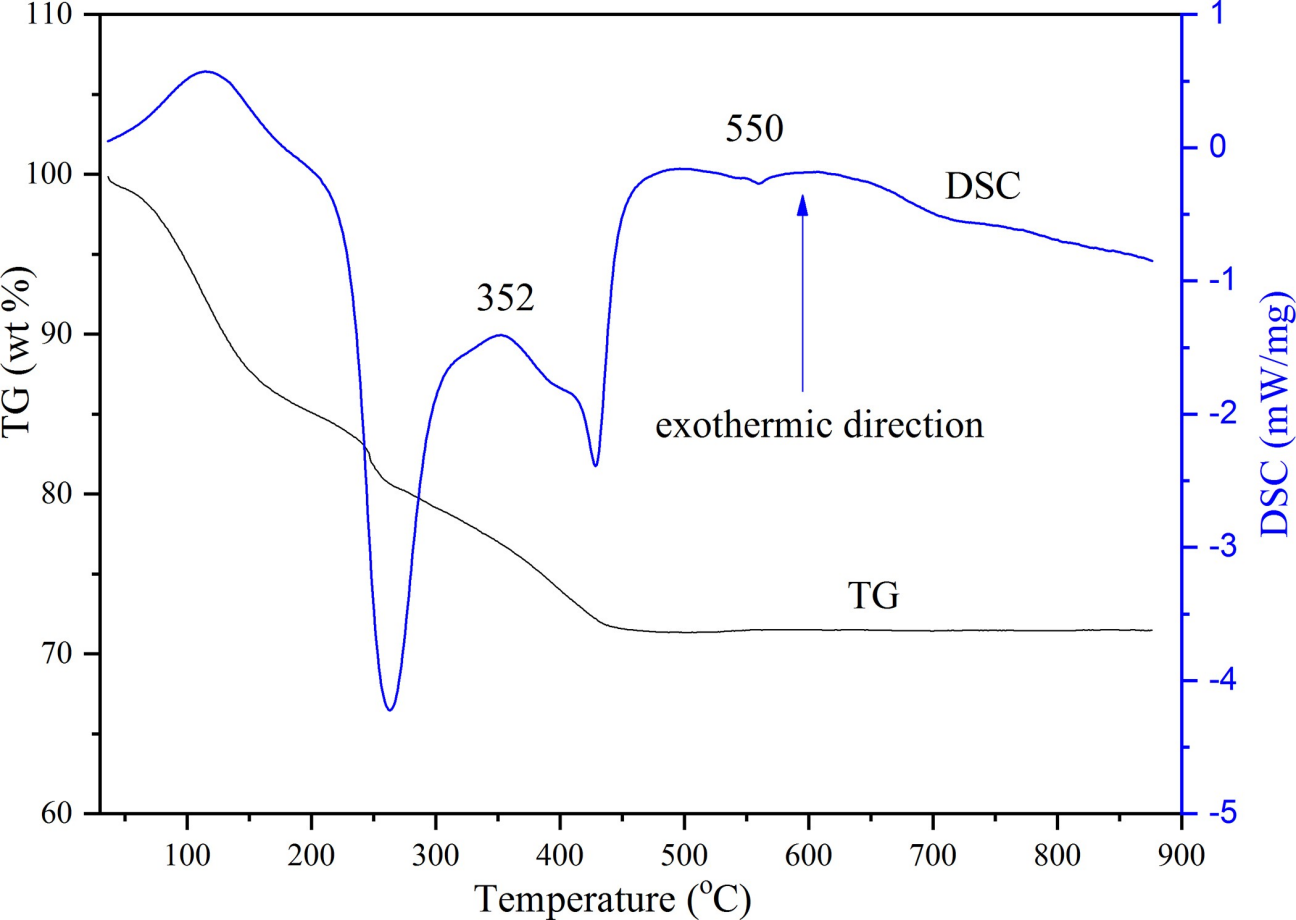
TG-DSC curves of the C/TiO_2_ catalyst.

To investigate the thermostability and components of the CT catalyst, thermogravimetric analysis was conducted. As it demonstrated in Fig 2, C/TiO_2_ catalyst shows an approximately 5% weight loss when heated to 100°C, suggesting water evaporation from the catalyst surface [30]. When it gets to 450°C, the weight loss was 29%, which could be attributed to the partial removal of tetrabutyl titanate hydrolysate bonded in the titanium dioxide. This instability of the sample indicated that C was successfully coated on the surface of TiO_2_ at the calculated temperature of 400°C. From the DSC curves, it is worth noting that a peak of an exothermic reaction appeared at approximately 352°C indicating a crystal transition of the catalyst from the amorphous to the anatase phase [30]. Crystal conversion is complete at 400°C, which is consistent with the previous XRD result.

The microstructures of the C/TiO_2_ and CuO_*x*_-C/TiO_2_ catalysts were characterized by SEM and TEM. Fig **A in S1 File** shows the SEM images of CT (before calcination). The sample is composed of many aggregated nanosized sphere particles. These spheres result from the hydrolysis of TBOT. To further observe the microstructure, the corresponding TEM images are presented in Fig B in **S1** File. The nanosized sphere particles have a distribution in average size of 200 to 300 nm, with smooth edges. From the HR-TEM (Fig B in **S1** File), layered amorphous structure on surfaces of the spheres are detected, which could be identified as carbon materials. It is noted that no lattice fringes were found in the catalyst, which suggests that the catalyst remains amorphous. The results are in accordance with the previous XRD pattern.

**Fig 3:**
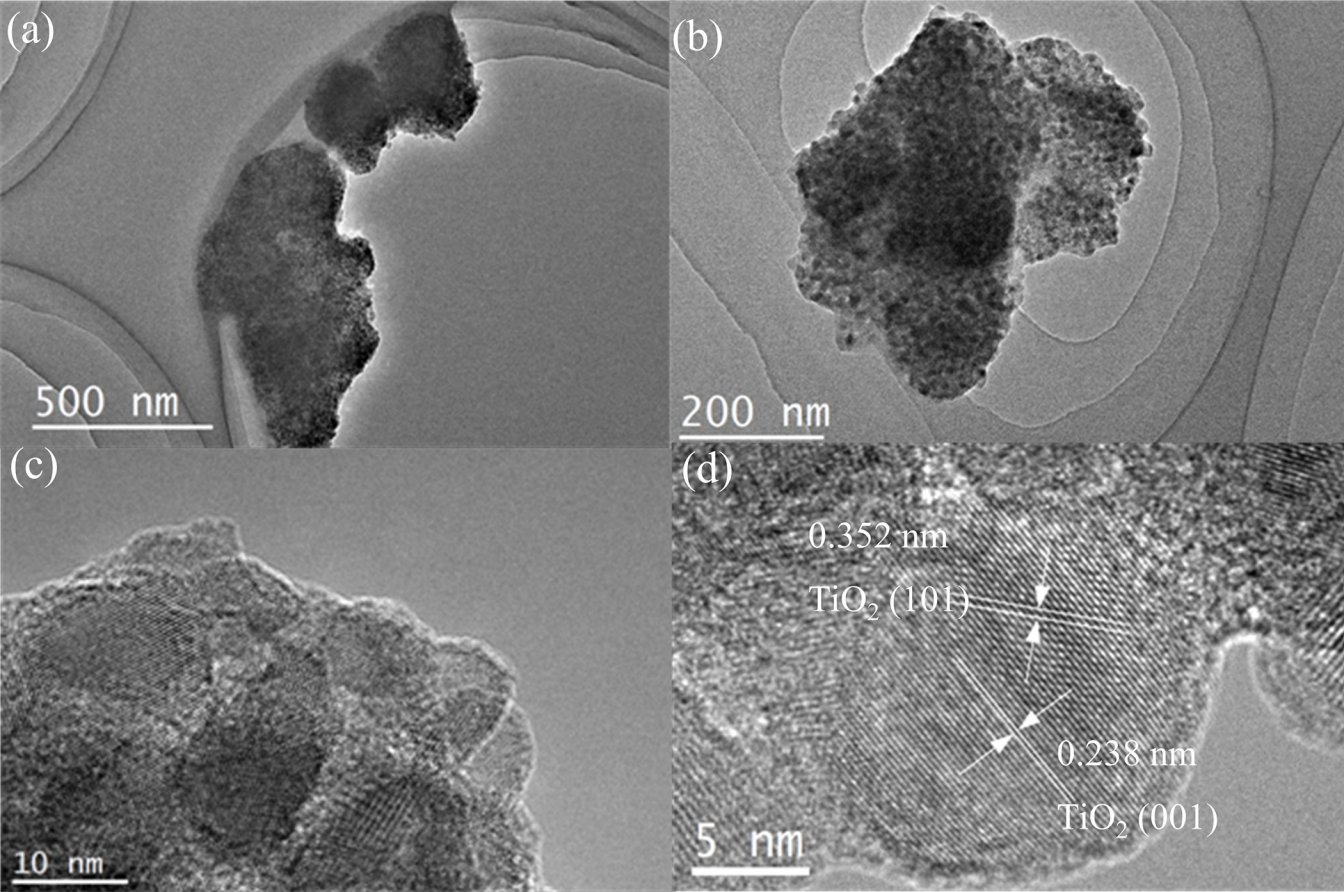
TEM (a, b) and HRTEM (c, d) images of CT400.

After heat treatment for 2 h, CT400 shows a similar SEM image with CT in Fig C in **S1** File. The spheres show a clear lattice fringe with spacings of 0.352 and 0.238 nm which match well with the 101 and 001 facet of anatase TiO_2_, respectively, as seen in Fig 3D [24]. The observed crystallite size of the spheres is approximately 10 nm, which is similar to the one calculated from the XRD results.

**Fig 4:**
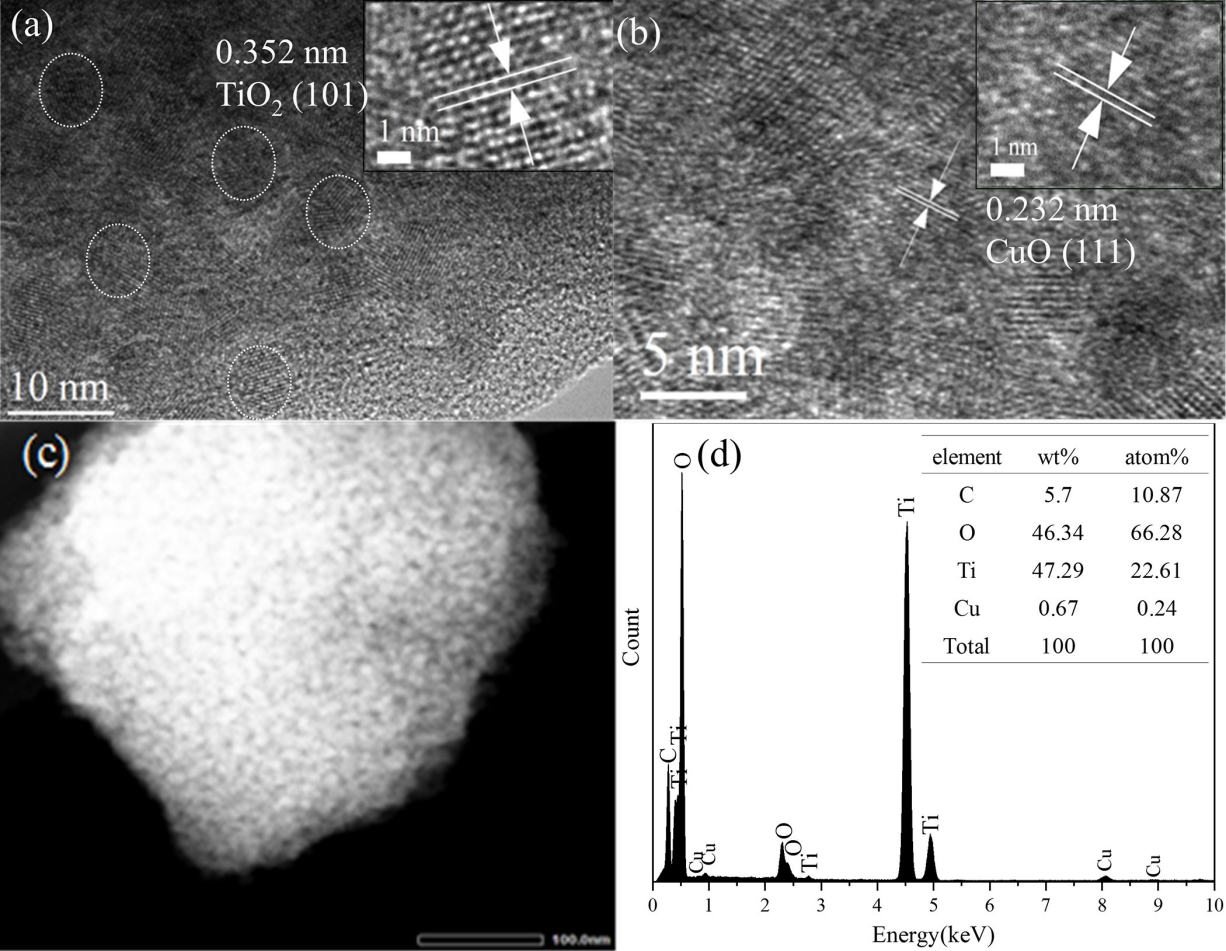
HRTEM images (a-b) of CuO_*x*_-CT400 and HAADF-STEM image (c) and EDX spectrum (d). The insets are the corresponding lattice fringes.

The SEM structure of CuO_*x*_-CT400 is presented in Fig D in **S1** File. A structure with surface porosity is observed. From the fractured surface structure, the catalyst inside are also full of nanosized particles. As part a and b of Fig 4 show, the HR-TEM images of CuO_*x*_-C/TiO_2_ clearly indicate 0.352 nm (101 facet of anatase, JCPDS 21–1272) [24] and 0.232 nm lattice fringes (111 facet of CuO, JCPDS 45–0937) [31]. The EDX spectra (Fig 4D) of the square area (Fig 4C) reveal that Cu, O, Ti, and C coexist in the catalyst. Furthermore, as displayed in the elemental mapping (Fig E in **S1** File), the individual elements of Cu, and C are uniformly distributed on the surface of TiO_2_.

**Fig 5:**
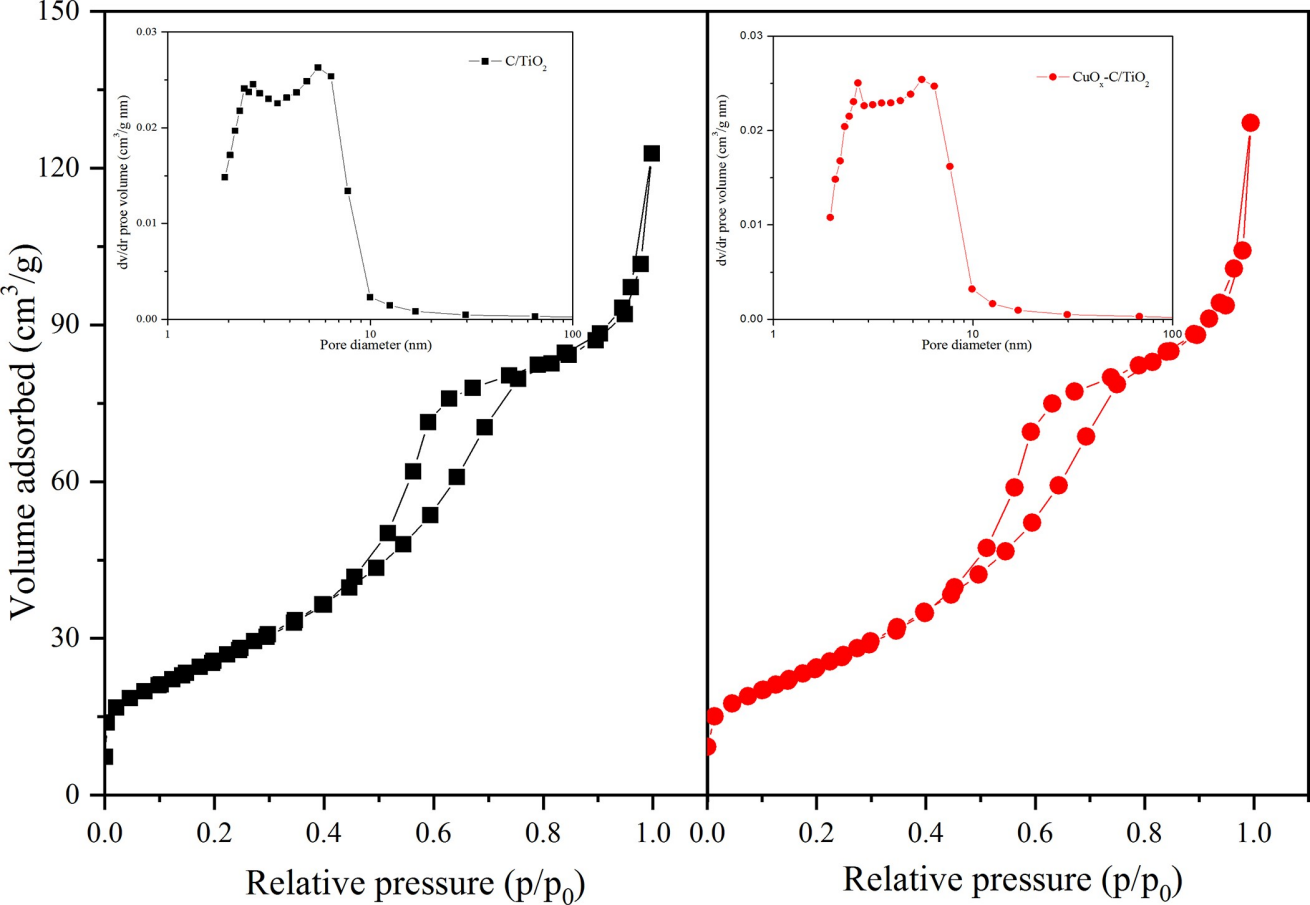
N_2_ adsorption–desorption isotherms for CT400 (a) and CuO_*x*_-CT400 (b) and corresponding pore size distribution spectra.

Fig 5 shows the nitrogen adsorption-desorption isotherms of CT400 and CuO_*x*_-CT400, both the catalysts exhibit type-IV patterns with H2 hysteresis, ascribed to a typical mesoporous structure [32]. After estimation with the Barret-Joyner-Halenda method, the pore size distribution is shown in the inset of Fig 5. The pore size is mainly in the range of 2.5 to 6.5 nm, which can be attributed to intra-agglomeration of primary particles. It is speculated that the TiO_2_ cluster was first formed during the hydrolysis of TBOT as the nucleus and then grown into larger particles with the framework of confined small mesopores [33]. The Brunauer–Emmett–Teller surface area and other parameters of CT400 as well as CuO_*x*_-CT400 are summarized in Table 1. The BET values only show a relatively mild decrease in the presence of Cu species, indicating a possible limited occlusion of the pores of the support by the presence of the co-catalyst.

**Table 1:** Model parameters of all the photocatalysts based on EIS and BET.

Samples	BET (m^2^/g)	Pore volume (mL/g)^a^	Pore diameter (nm)^a^	R_s_(Ω/cm^2^)^b^	R_ct_(Ω/cm^2^)^c^	C_dl_ × 10^8^ (F/cm^2^)^d^	Z_w_ × 10^3^ (S^0.5^/Ω cm^2^)^e^
**CT400**	97.3	0.216	6.14	26.2 ± 1.8	122 ± 2.0	0.267 ± 0.088	9.90 ± 0.55
**CuO**_***x***_**-CT400**	92.7	0.228	6.57	27.3 ± 0.4	80.6 ± 1.4	10.5 ± 0.30	7.89 ± 0.61

^a^Determined by the BJH method using the nitrogen absorption branch of the isotherm.

^b^The reactivity resistance.

^c^The solution resistance.

^d^The double-layer capacitance.

^e^The Warburg impedance.

**Fig 6:**
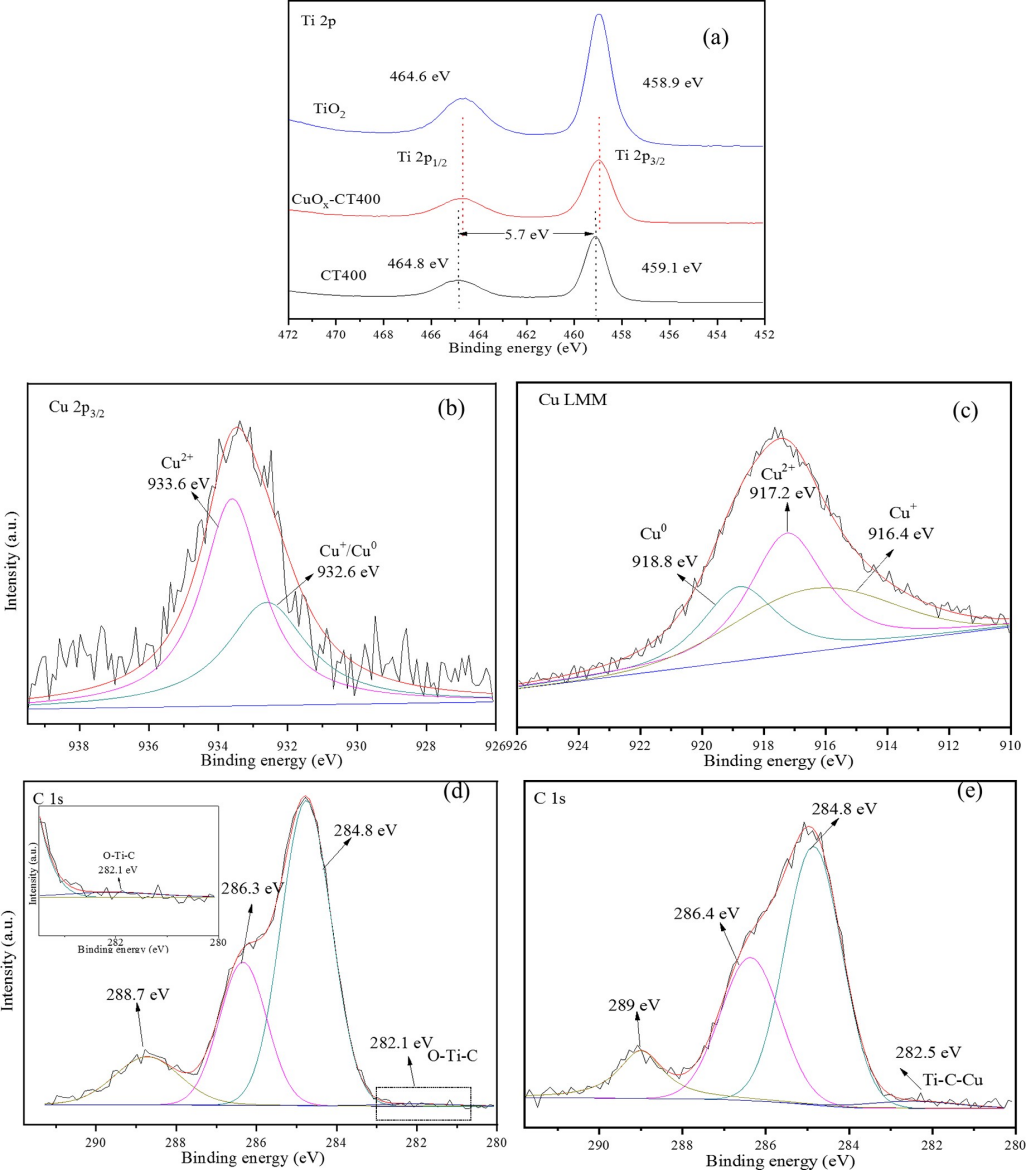
High-resolution XPS spectra of Ti 2p and Cu 2p for CuO_x_-CT400 (a, b), Cu LMM spectra for CuO_*x*_-CT400 (c) and C 1s spectra for CT400 (d) and CuO_*x*_-CT400 (e).

Fig 6 shows the XPS spectra of CT400 and CuO_*x*_-CT400. In Fig 6A, the binding energy peaks at 459.1 eV and 464.8 eV of CT400 correspond to the Ti 2p_3/2_ and Ti 2p_1/2_ states with a gap of 5.7 eV, which suggests the presence of the typical Ti^4+^ state [34]. Compared with the previously prepared TiO_2_ sample [22] (the Ti 2p3/2 state is at 458.9 eV and the Ti 2p_1/2_ state is at 464.6 eV), the CT400 shows a slight 0.2 eV shift to higher binding energy. The result may suggest the surface interaction between the carbonaceous species with the TiO_2_ surface. The Ti 2p binding energy of CuO_*x*_-CT400 shifts to lower binding energy in contrast with CT400, which indicates charge transfer between the Cu species and C/TiO_2_ [31]. The high resolution spectra of Cu 2p_3/2_ for CuO_*x*_-CT400 (Fig 6B) exhibit two characteristic peaks at 933.6 and 932.6 eV with XPS-peak-differentiation-imitating analysis. The 933.6 eV peak is attributed to the Cu^2+^ state while the 932.6 eV could be Cu^+^ or Cu^0^ [35]. To further confirm the exact state of Cu species, the Cu LMM spectra were obtained through AES characterization, as shown in Fig 6C. The broad and asymmetrical Cu LMM spectrum suggests the presence of one more component. After peak fit processing, the three characteristic peaks are 917.2, 916.4, and 918.8eV, corresponding to Cu^2+^, Cu^+^, and Cu^0^, respectively [35]. The modified Auger parameter (α’) is also a very useful indicator to distinguish between Cu^+^ and Cu^0^ and is calculated as follows [36]:

$$\alpha ’\;\left(eV\right)=BE\;\left(Cu\;2p_{3/2}\right)+KE\;\left(Cu\;LMM\right)$$

With this calculation, the values of α’ are 1850.8 eV, 1849 eV and 1851.4 eV corresponding to Cu^2+^, Cu^+^, and Cu^0^, respectively.

Fig 6D and 6E shows the high resolution C1s spectra of CT400 and CuO_*x*_-CT400. Three evident peaks at 284.8, 286.3, and 288.7 eV are detected for CT400. The C1s peak at 284.8 eV originated from sp^2^ hybridized carbon [37]. The peaks at 286.3 eV and 288.7 eV appear in sample CT400, which are attributed to the oxygen bound species C–O and Ti–O–C, respectively [34, 38]. The peak at 282.1eV with low intensity is close to the C 1s peak related to the binding energy between Ti and C (281.8 eV). Only small amount of C doped into the inner TiO_2_ lattice, other carbon may exist as the carbonaceous species formed at the surface of TiO_2_ to serve as a conductor. After loading the Cu species, the CuO_*x*_-C/TiO_2_ catalyst also exhibits the similar peak shape but a small peak at 282.5 eV, which is close to the Ti-C bond [39] and shifts to higher binding energy, deduced as the Ti-C-Cu bond. Based on the above, it can be concluded that Cu species could be grafted on the carbonaceous materials.

### 3.2 Optical properties

**Fig 7:**
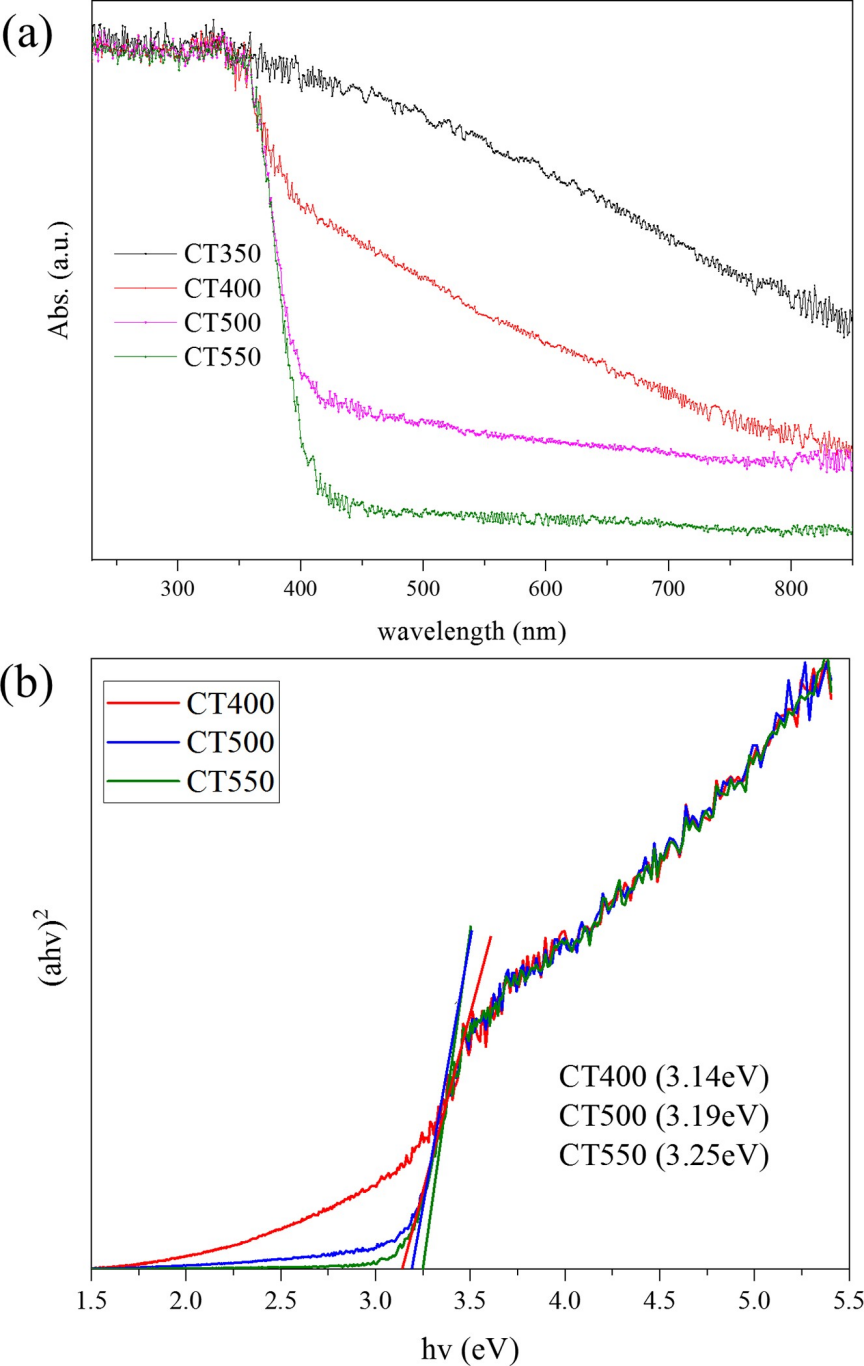
UV-vis diffuse reflectance spectra (a) and the band gap evaluation for linear dependence of (αhv)^2^ versus hv (b) for the samples.

UV-vis diffuse reflectance spectroscopy (DRS) was performed on CT350, CT400, CT500, and CT550 to determine the light harvesting, and the results are shown in Fig 7. In Fig 7A, light harvesting ability exhibits an evident change with different calcination temperatures. Compared to pure TiO_2_, the absorption edge of CT350 obviously shifts to the visible light region, which can be attributed to the photo absorption property of the carbonaceous materials absorbed on the TiO_2_ surface [40]. The visible absorption of C/TiO_2_ decreased significantly with increasing calcination temperature. CT550 exhibits a similar spectrum to the pure TiO_2_ lattice. This result proves that carbonaceous species were gradually removed due to the higher temperature, which lead to the decreased absorption of visible light. Notably, the spectra of CT350 exhibit DRS spectra similar to those reported in the literature [38], in which the carbon is coated on the TiO_2_ surface. Based on these results, it can be speculated that carbonaceous materials in the CT catalyst mainly adsorb on the TiO_2_ surface, which is in accordance with the XPS analysis. As shown in Fig 7B, the direct band gaps of CT400, CT500, CT550 are estimated to be 3.14, 3.19, 3.25 eV, respectively, by using a Tauc plot. They are slightly lower or higher than the reported values of pure anatase TiO_2_ [41], which could be attributed to surface defects such as small amounts of carbon impurities [42].

**Fig 8:**
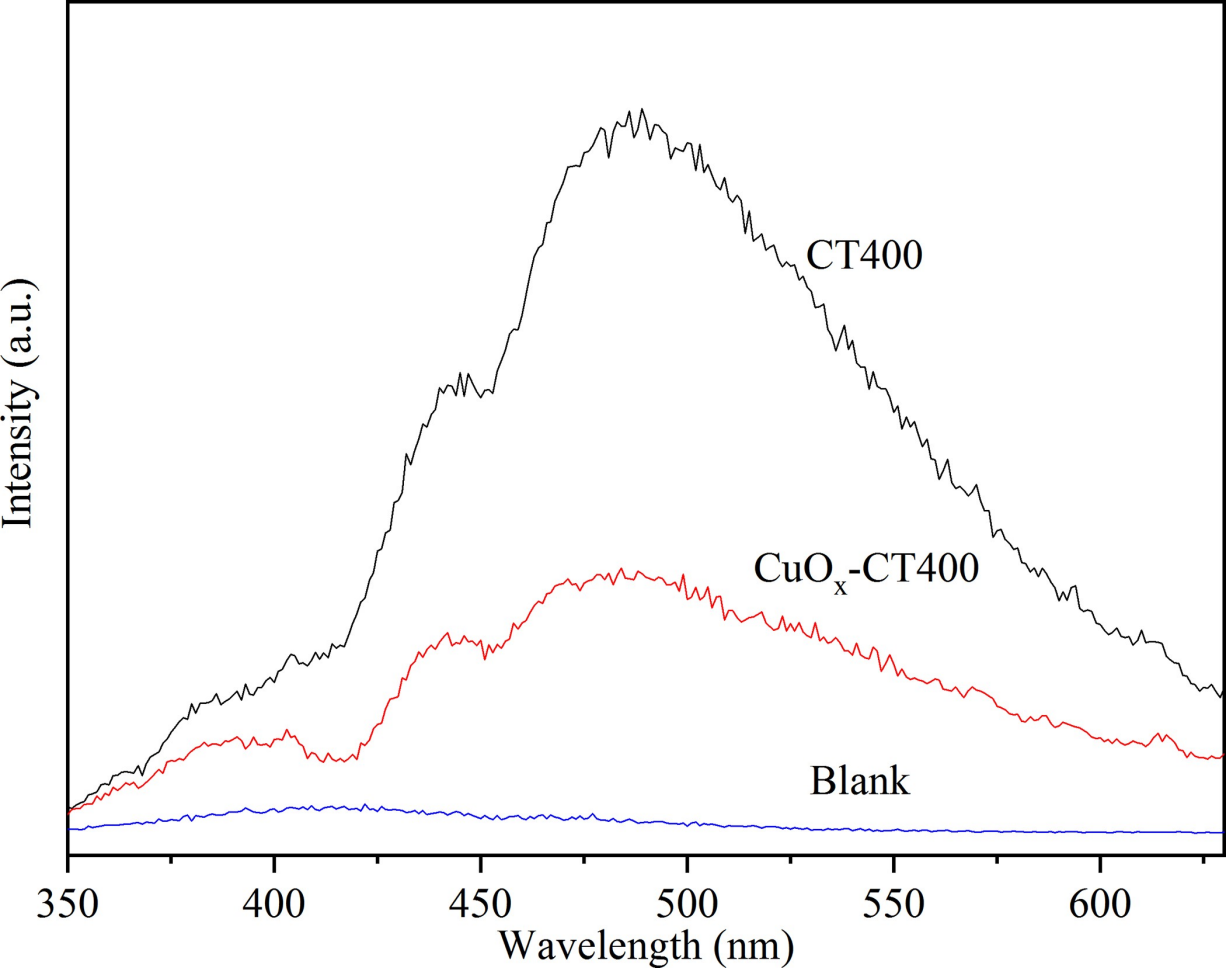
PL spectra of CT400 and CuO_*x*_-CT400.

To explore the charge carrier transfer and recombination, photoluminescence emission spectra were employed. As shown in Fig 8, the two curves exhibit similar shapes but vary in fluorescence intensity. There are three peaks (408, 450, and 483 nm) in the wavelength range of 350–600 nm. The peak of 408 nm is attributed to the interbands PL phenomenon. The two peaks at 450 and 483 nm are ascribed to the band edge free excitons [43]. Overall, the PL intensity decreases with the loading of CuO_*x*_, which indicates that CuO_*x*_ favors the transfer of photo-induced electrons and restrains the recombination of photo-induced electrons and holes [44].

### 3.3 Photoelectrochemical properties

**Fig 9:**
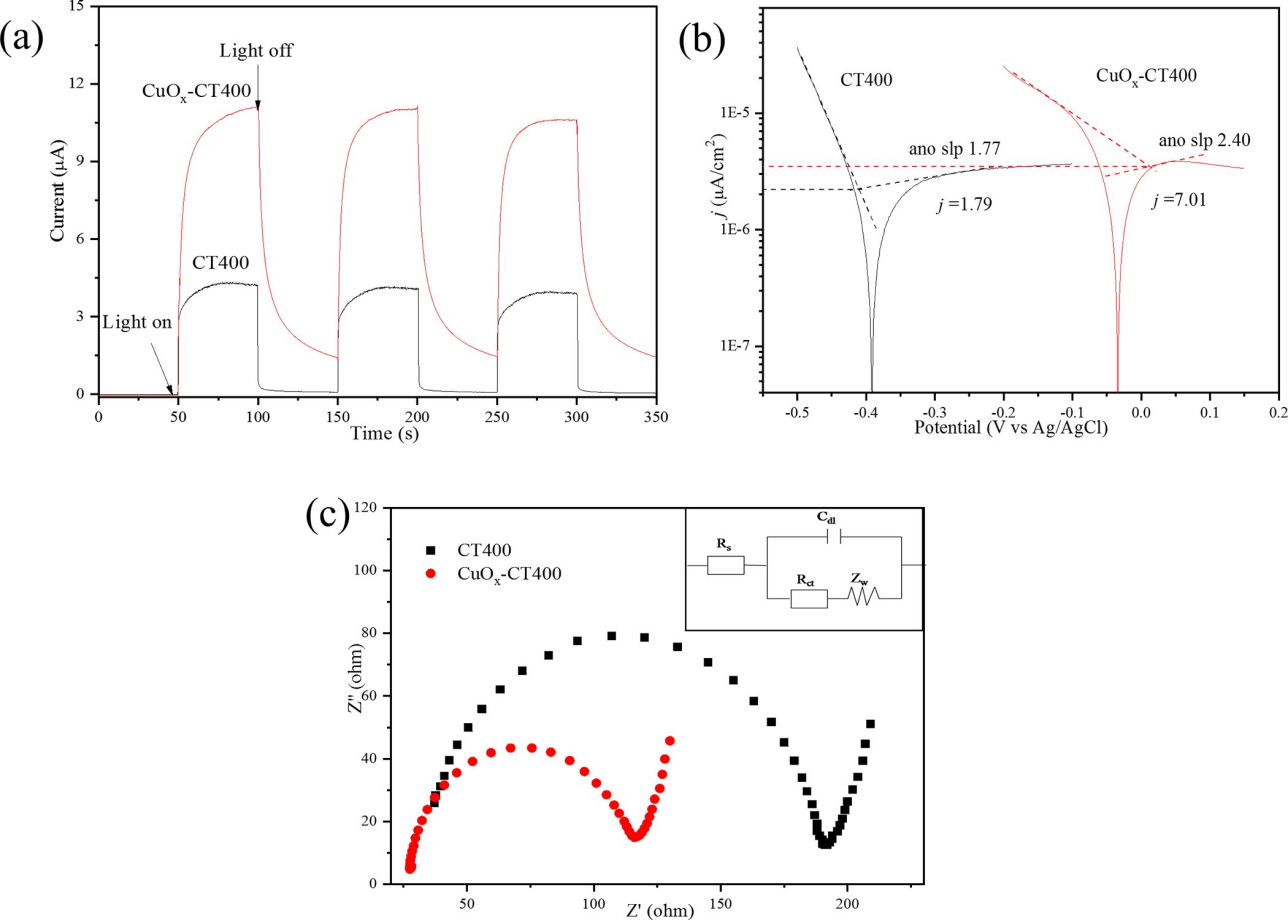
Photoelectrochemical properties of CT400 and CuO_*x*_-CT400 catalyst with i-t spectra (a), Tafel spectra (b), and Nyqusit spectra (c). The inset is the equivalent circuit model.

Fig 9 shows the photoelectrochemical properties of the CT400 and CuO_*x*_-CT400 catalysts. The experiment was conducted to reveal the photoexcited electron transfer. As Fig 9A shows, the transient photocurrent increases instantly with the light on and decreases with the light off. The CuO_*x*_-CT400 catalyst exhibits higher photocurrent up to 11.3 μA compared to CT400 with 4.4 μA. Generally, the higher transient photocurrent indicates more electron transfer [45]. It is worth noting that the CuO_*x*_-CT400 catalyst shows an evident spectrum trail when the light is off. The phenomenon is ascribed to complex photoelectron transfer involving the valence state change of Cu species.

Fig 9B shows the Tafel spectra of both CT400 and CuO_*x*_-CT400. Anode slope and corrosion current (j) are two major parameters of Tafel characterization. The anode slopes are 1.77 and 2.40 and the corrosion currents are 1.79 and 7.01 μA/cm^2^ for CT400 and CuO_*x*_-CT400, respectively. The larger anode slope and j values suggest the faster photoelectron transfer [46] of CuO_*x*_-CT400, compared with CT400.

To further test the photoexcited charge carries transfer resistance of the catalyst, electrochemical impedance spectroscopy (EIS) [47] methods were used, and the results are shown in Fig 9C. The Nyquist spectra exhibit a high-frequency half-circle and a low-frequency straight line, which is consistent with the reported literature. An equivalent circuit model shown in the image was constructed by ZsimpWin 3.10d based on the spectra. The model includes four parameters, R_ct_ (the reactivity resistance), R_s_ (the solution resistance), C_dl_ (double-layer capacitance), and Z_w_ (Warburg impedance), as shown in Table 1. Generally, R_ct_ indicates the resistance of the charge carrier transfer. Notably, R_ct_ values for CT400 (122 ± 1.69 Ω/cm^2^) decreased to 80.6 ± 1.4 Ω/cm^2^ for CuO_*x*_-CT400. The result indicates that CuO_*x*_ modification significantly contributes to making the photoexcited charge carrier transfer more favorable and faster [48].

### 3.4 Photocatalytic activity

**Fig 10:**
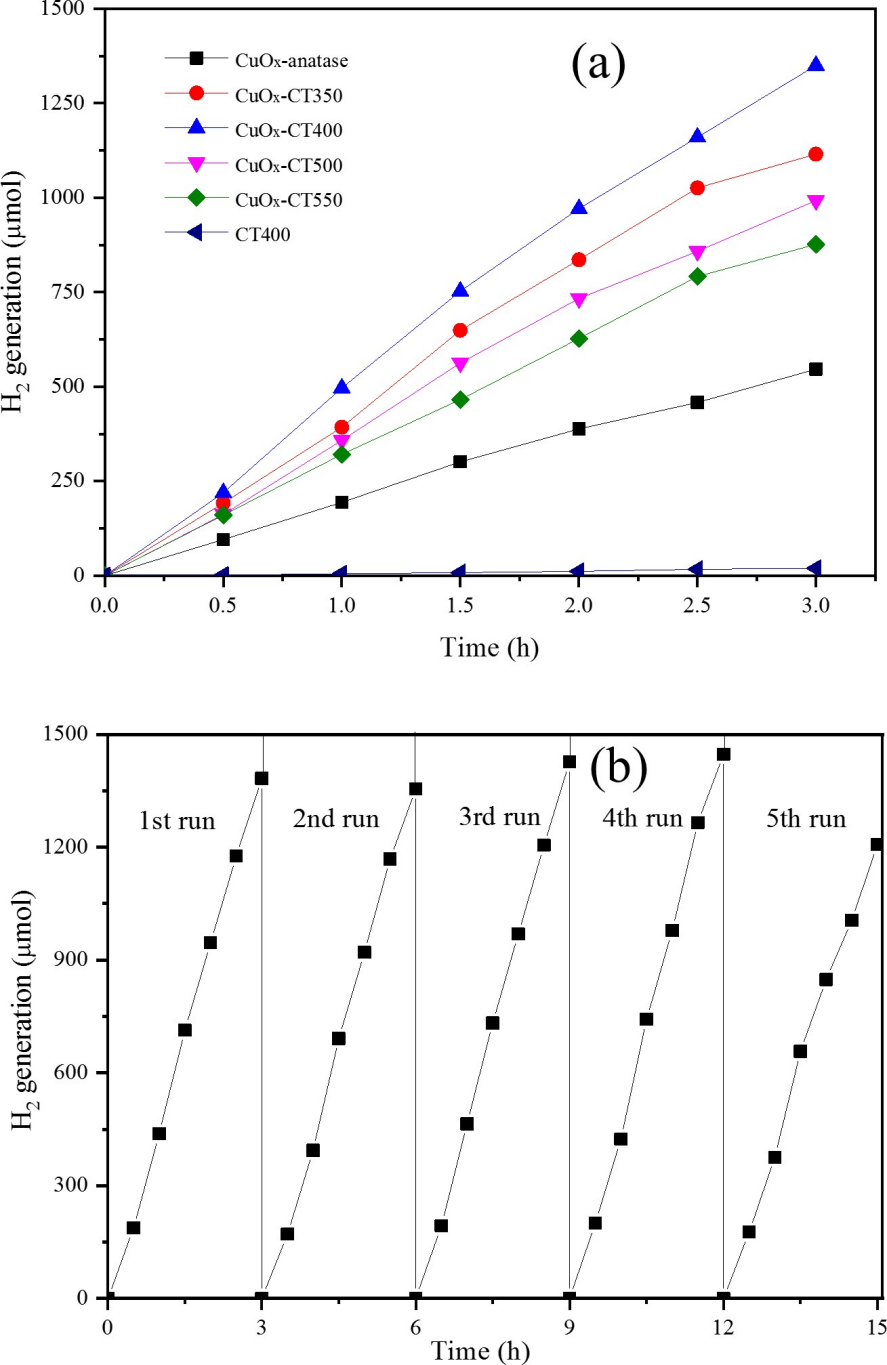
Photocatalytic activity experiment (a) and cycle experiment for CuO_*x*_-CT400 (b).

Fig 10A shows the photocatalytic hydrogen evolution of prepared samples under UV-visible light. The H_2_ evolution amount follows the order of CuO_*x*_-CT400 > CuO_*x*_-CT350 > CuO_*x*_-CT500 > CuO_*x*_-CT550 > CuO_*x*_-anatase > CT400. The CuO_*x*_-CT400 catalyst exhibits a high hydrogen generation rate of 433.3 μmol/h, which reaches 56 times that of CT400 (7.7 μmol/h). CuO_*x*_-anatase was prepared for comparison since it is difficult to prepare CuO_*x*_-TiO_2_ without carbon according to this method (the precursor Ti(OBu)_4_ was both a carbon source and a titanium source). The H_2_ evolution efficiency with CuO_*x*_-anatase is 216.7 μmol/h, about half that of with CuO_*x*_-CT400. The calcination temperature plays an important role for the H_2_ generation of CuO_*x*_-CTR catalysts. Generally, the higher temperature can result in higher crystallinity and lower carbonaceous material content of the catalyst. However, 400°C is the optimal temperature indicating that crystallinity is not the main factor for the photoactivity. It can be speculated that carbon content and crystalline type are associated with photocatalytic H_2_ generation.

The cycle experiment of the hydrogen evolution of CuO_*x*_-CT400 is presented in Fig 10B. The H_2_ generation is always high in the first four cycles and shows a little decrease in the 5th run.

The CO_2_ and CO production of CT400 and CuO_*x*_-CT400 are shown in Fig F in **S1** File. A significantly enhanced CO_2_ and CO production rate was observed between CT400 and CuO_*x*_-CT400, which is consistent with the H_2_ evolution rate.

### 3.5 Mechanism

The results of the photoactivity experiment reveal that the carbon coated on the CuO_*x*_-CT samples is important for the elevated photoactivity. However, the CT400 catalyst without Cu modification exhibited low photocatalytic efficiency. It seems reasonable to deduce that carbon materials could act as good electron conductors instead of proton reduction sites. Moreover, Joo et al. [49] prepared four different carbon-TiO_2_ catalysts and concluded that electrically conductive carbon could only facilitate charge carries separation but made no direct contribution to the hydrogen formation. Further, in the synthesized materials, CuO_*x*_ species can be crucial co-catalysts which serve as both electron acceptor and surface redox reaction sites. Therefore, the carbonaceous material is speculated to act as an electron-bridge providing a new electron transfer channel from TiO_2_ to the CuO_*x*_ species.

**Fig 11:**
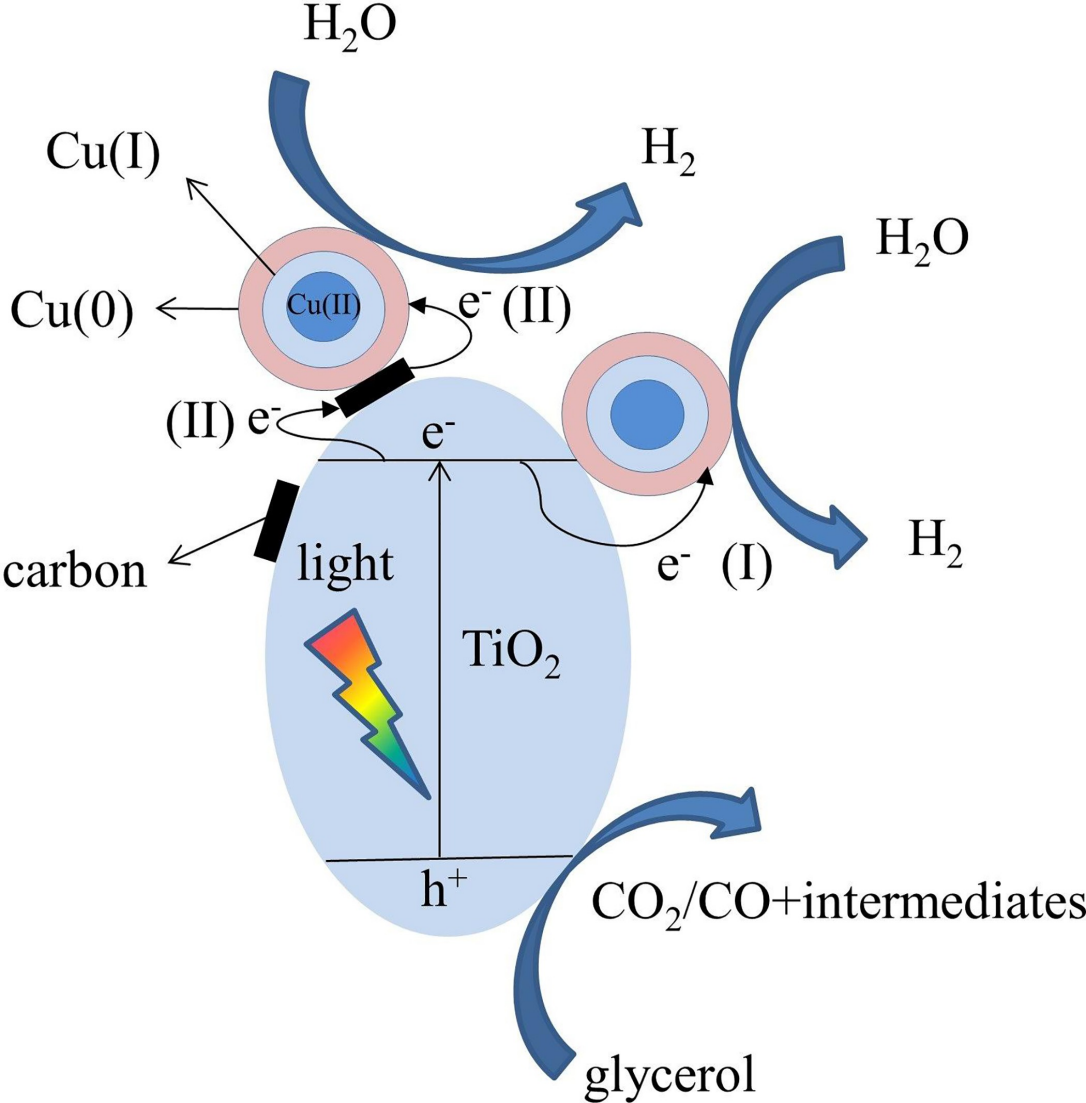
Photocatalytic hydrogen evolution mechanism of the CuO_*x*_-C/TiO_2_ catalyst with direct (I) and indirect (II) electron transport pathways.

According to the results observed and discussed above, the probable mechanism of CuO_*x*_ modified C/TiO_2_ for photocatalytic H_2_ evolution featuring a synergistic effect of CuO_*x*_ and carbon is presented in Fig 11. Structurally, carbonaceous materials are coated on the TiO_2_ surface through Ti-O-C bonds and CuO_*x*_ species can graft on both carbon materials and the TiO_2_ surface. Electrons were photoexcited to the conduction band (CB) of TiO_2_ with holes staying in the valence band (VB) under UV-visible light irradiation (Eqs 1 and 2). The electrons on the CB can go through two pathways. In pathway I, the electrons can be caught by the CuO_*x*_ species to form Cu(I) or Cu(0), which results in efficient electron-hole separation (Eq 3). Meanwhile, CuO_*x*_ species can act as proton reduction reaction sites for hydrogen generation (Eq 4). In pathway II, the CB electrons first transfer to the carbon material and finally reach the CuO_*x*_ species. The new pathway enhanced the CuO_*x*_ effect as the co-catalyst by providing a new electron transfer route to promote the charge carrier separation.

Accordingly, the hydrogen evolution rate is significantly boosted by the synergistic effect of CuO_*x*_ and carbon materials over the CuO_*x*_-C/TiO_2_ catalyst. Glycerol could first be adsorbed onto the surface of TiO_2_ and then was oxidized to CO_2_, CO, and other intermediates (C_2_H_4_O_2_, C_2_H_2_O_3_, C_2_H_4_O_3_, C_3_H_6_O_3_, C_3_H_6_O_4_, etc.) [22] by the photoexcited holes or hydroxyl radicals (Eqs 5 and 6).

$$\mathrm{Catalyst}\;\overset{\mathrm{light}}{\to }\;h_{\mathrm{VB}}^{+}+e_{\mathrm{CB}}^{‐}$$ (1)

$$2H^{+}+2e_{\mathrm{CB}}^{‐}\;\to \;H_{2}$$ (2)

$$\mathrm{Cu}(\mathrm{II})+e_{\mathrm{CB}}^{‐}\;\to \;\mathrm{Cu}(I)/\mathrm{Cu}(0)$$ (3)

$$2\mathrm{Cu}(I)/\mathrm{Cu}(0)+{2H}^{+}\;\to \;2\mathrm{Cu}(\mathrm{II})+H_{2}$$ (4)

$$h_{\mathrm{VB}}^{+}+{\mathrm{OH}}^{‐}\;\to \;●\mathrm{OH}$$ (5)

$$\mathrm{Glycerol}+h_{\mathrm{VB}}^{+}/●\mathrm{OH}\;\to \;\mathrm{intermediates}\;\to \;CO_{2}/\mathrm{CO}+H_{2}O$$ (6)

## 4. Conclusion

Carbon coated on a TiO_2_ surface modified with CuO_*x*_ has been prepared via a simple hydrolysis and photo-reduction method. With systematic structure characterization, carbonaceous materials were observed to be absorbed onto the surface of TiO_2_ nanoparticles. The Cu species existed as CuO_*x*_ with multivalence states grafted both on the TiO_2_ surface and the carbonaceous materials. With CuO_*x*_ functioning as a cocatalyst, the CuO_*x*_-CTR exhibited high activities in photocatalytic hydrogen evolution. The photocatalytic hydrogen generation rate of the CuO_*x*_-CT400 catalyst (433.3 μmol/h) is 56 times as high as that of CT400 (7.7 μmol/h). This significant increase is attributed to the synergistic effect between CuO_*x*_ and the carbon species. CuO_*x*_ grafted on the surface of the catalyst accepts photoexcited electrons inhibiting electron-hole recombination and serving as proton reduction sites. Meanwhile, carbonaceous materials function as good conductors by transferring electrons to the CuO_*x*_, which significantly enhances the CuO_*x*_ co-catalyst effect. This work demonstrates a simple method to prepare a Cu and C co-modified TiO_2_ catalyst with high photocatalytic performance and proposes the synergistic effect between copper and carbon, which presents a significant step in determining the photoexcited electron transfer mechanism.

## Supporting information

S1 File **Table A in S1 File.** Comparison of various TiO_2_-based photocatalysts. **Fig A in S1 File.** SEM images of CT. **Fig B in S1 File.** TEM (a–c) and HRTEM (d) images of CT. **Fig C in S1 File.** SEM images of CT400. **Fig D in S1 File.** SEM images of CuO_*x*_-CT400. **Fig E in S1 File.** Elemental mapping of CuO_*x*_-CT400 (a-d) for C, Cu, O, and Ti. **Fig F in S1 File.** CO_2_ (a) and CO (b) production of CT400 and CuO_*x*_-CT400. Additionally, the raw data for figures has been uploaded to Figshare (http://dx.doi.org/10.6084/m9.figshare.7951724).
(DOCX)
