# Supplementary material for: Enhanced hydrogen evolution from CuOx-C/TiO2 with multiple electron transport pathways
Source: PLoS One. 2019 Apr 15;14(4):e0215339. doi: 10.1371/journal.pone.0215339 (PMC6464221; doi:10.1371/journal.pone.0215339)
Supplement: S1 File — Table A in S1 File. Comparison of various TiO2-based photocatalysts. Fig A in S1 File. SEM images of CT. Fig B in S1 File. TEM (a–c) and HRTEM (d) images of CT. Fig C in S1 File. SEM images of CT400. Fig D in S1 File. SEM images of CuOx-CT400. Fig E in S1 File. Elemental mapping of CuOx-CT400 (a-d) for C, Cu, O, and Ti. Fig F in S1 File. CO2 (a) and CO (b) production of CT400 and CuOx-CT400. Additionally, the raw data for figures has been uploaded to Figshare (http://dx.doi.org/10.6084/m9.figshare.7951724). (DOCX) [file pone.0215339.s001.docx]

Appendix A. Supplementary data for

Enhanced hydrogen evolution from CuO*_x_*-C/TiO_2_ with multiple electron transport pathways

Xiuying Huang, Meng Zhang, Runze Sun, Gaoyuan Long, Yifan Liu, and Weirong Zhao*

Department of Environmental Engineering, Zhejiang University, Hangzhou 310058, China

Number of pages (including cover page): 6

Number of Table: 1

Number of Figures: 6

^*^Corresponding author

Tel.: +86-571-8898-2032; fax: +86-571-8898-2032.

E-mail address: wrzhao@vip.163.com (Weirong Zhao)

Table A Comparison of various TiO_2_-based photocatalysts

| **Catalyst** | **Light source** | **Organic scavenger** | Application | **Author** | **Reference** |
| --- | --- | --- | --- | --- | --- |
| Pt/TiO_2_ | λ=325 nm | Ethanol | H_2_ generation  (19.3μL_H2_h^-1^) | [JeongEun](https://pubs.acs.org.ccindex.cn/author/Yoo%2C+JeongEun) | The Journal of Physical Chemistry C, 2016, 120, 29, 15884-15892 |
| M ^n+^/TiO_2_ (M=Cu; Fe; Ni; Cr) | UV-Vis | None | decomposition of acetaldehyde | Murakami | Applied Catalysis A: General, 2008, 348, 1, 148-152 |
| Cu(II)/TiO_2_ | λ=470 nm | None | decomposition of acetaldehyde | Nosaka | The Journal of Physical Chemistry C, 2015, 115, 43, 21283-21290 |
| quantum Cu(II) nanodots/ TiO_2_ | UV-Vis | Glycerol | H_2_ generation (1470μmol.h^-1^ g^-1^) | Zhang | The Journal of Physical Chemistry C, 2016, 120, 20, 10746-10756 |
| graphene/TiO_2_ | UV-Vis | Methanol | H_2_ generation  (736μmol.h^-1^ g^-1^) | Xiang | Nanoscale, 2011, 3, 3670-3678 |
| TiO_2_/RGO/Cu(II) | λ=470 nm | None | Photodegradation of phenol | Zhang | ACS Applied Materials & Interfaces, 2015, 7, 3, 1816-1823 |
| Cu_2_O–TiO_2_/rGO | UV-Vis | Glycerol | H_2_ generation (110 968 μmol.h^−1^ g_cat_^−1^) | Babu | Nanoscale, 2015,7, 7849-7857 |
| CuO_x_-C/TiO_2_ | UV-Vis | Glycerol | H_2_ generation | Huang | this work |


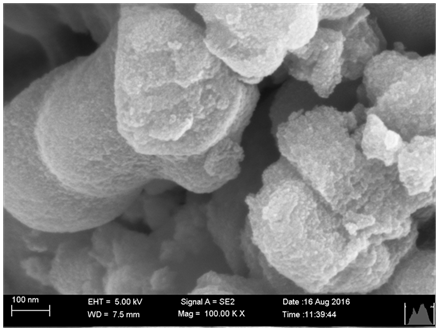

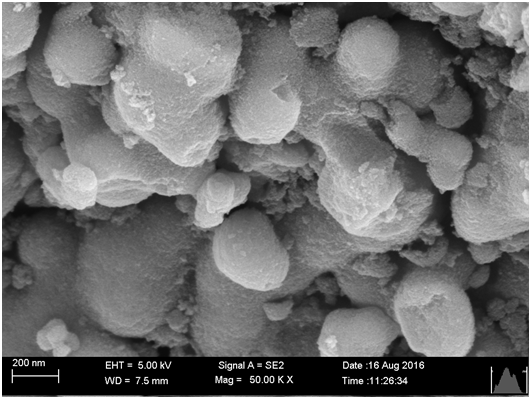

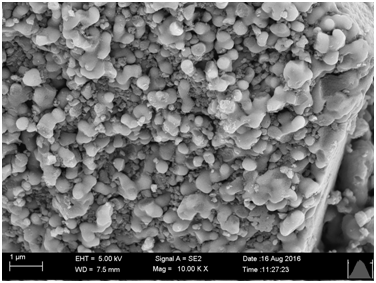

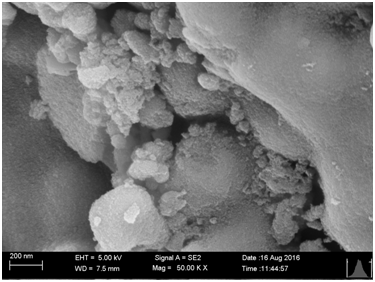


(a)

(b)

(d)

(c)

Fig. A SEM images of CT


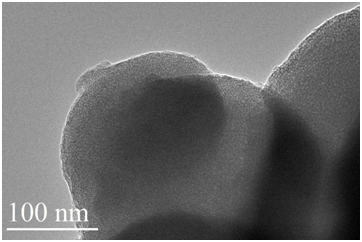

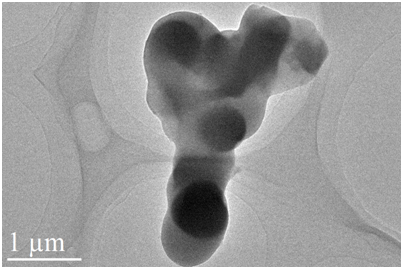

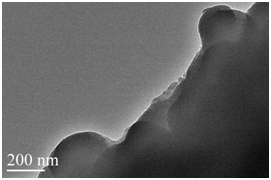

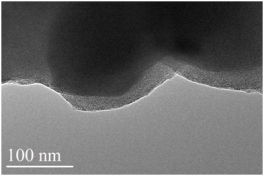


(b)

(c)


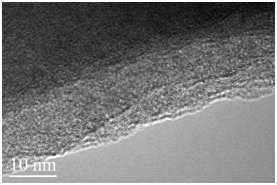


(d)

(a)

Fig. B TEM (a–c) and HRTEM (d) images of CT


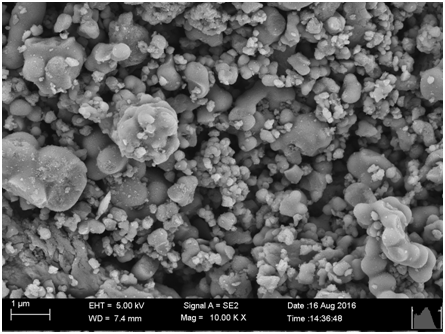

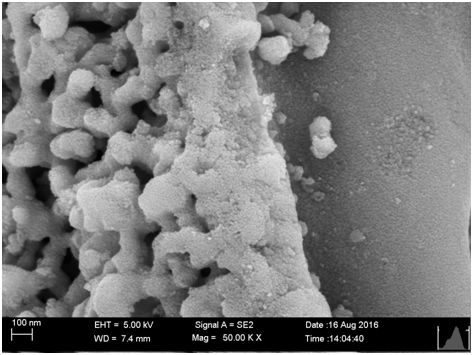

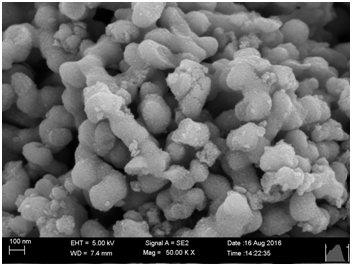

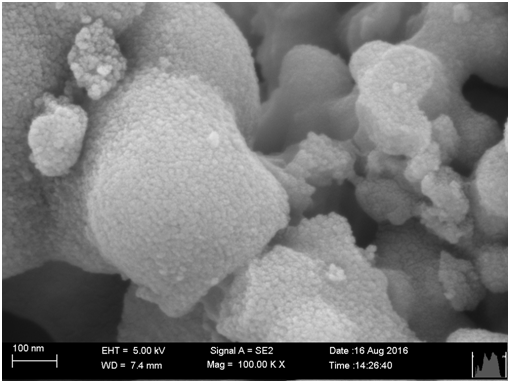


(a)

(b)

(c)

(d)

Fig. C SEM images of CT400


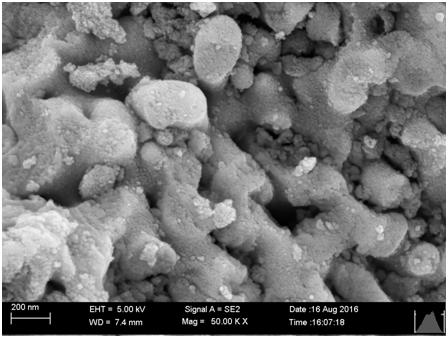

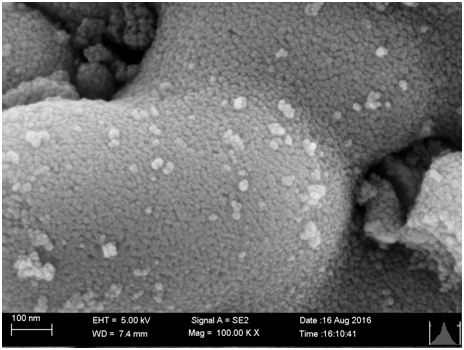

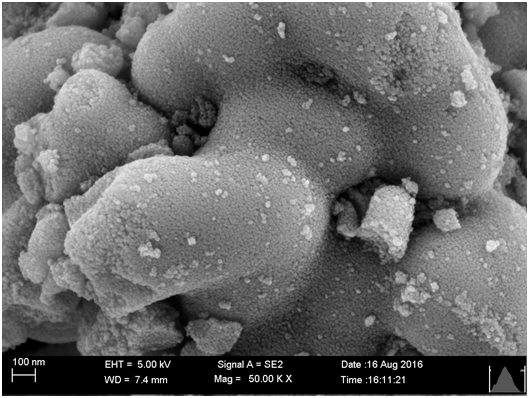

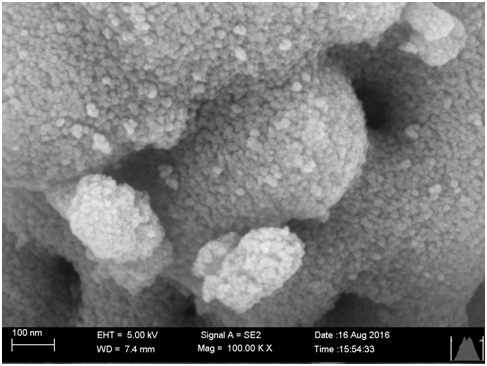


(a)

(b)

(c)

(d)

Fig. D SEM images of CuO*_x_*_­_-CT400


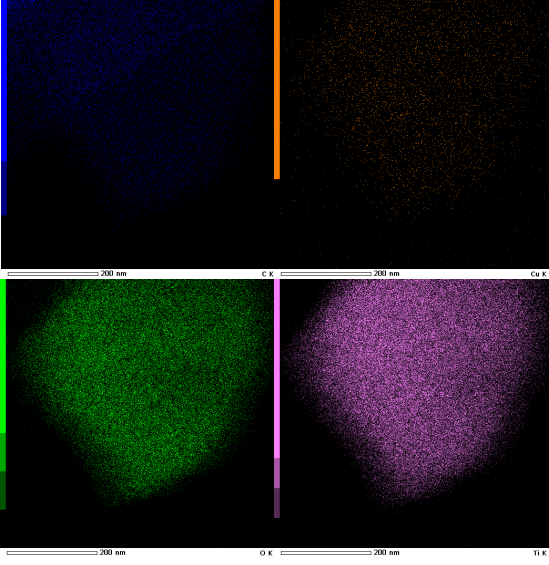


Fig. E Elemental mapping of CuO_x­_-CT400 (a-d) for C, Cu, O, and Ti

(a)

(b)

Fig. F CO_2_ (a) and CO (b) production of CT400 and CuO*_x_*-CT400
